# Accuracy Assessment of Semi-Automatic Measuring Techniques Applied to Displacement Control in Self-Balanced Pile Capacity Testing Appliance

**DOI:** 10.3390/s18114067

**Published:** 2018-11-21

**Authors:** Zbigniew Muszyński, Jarosław Rybak, Paulina Kaczor

**Affiliations:** 1 Faculty of Geoengineering, Mining and Geology, Wrocław University of Science and Technology, 50-370 Wrocław, Poland; paulina.kaczor@pwr.edu.pl; 2 Faculty of Civil Engineering, Wrocław University of Science and Technology, 50-370 Wrocław, Poland; jaroslaw.rybak@pwr.edu.pl

**Keywords:** vertical displacements, motorized total station, LIDAR, terrestrial laser scanning, bi-directional static load test

## Abstract

Static load tests of foundation piles are the basic method for the designing or verification of adopted design solutions which concern the foundation of a building structure. Preparation of a typical test station using the so-called inverted beam method is very expensive and labor-intensive. The settlement values of the loaded pile are usually recorded using accurate dial gauges. These gauges are attached to a reference beam located in close proximity to the pile under test, which may cause systematic errors (difficult to detect) caused by the displacement of the adopted reference beam. The application of geodetic methods makes it possible to maintain an independent, external reference system, and to verify the readouts from dial gauges. The article presents an innovative instrumentation for a self-balanced stand for the static load test made from a closed-end, double steel pipe. Instead of typical, precise geometric leveling, the semi-automatic measuring techniques were used: motorized total station measurement and terrestrial laser scanning controlled by a computer. The processing of the acquired data made it possible to determine the vertical displacements of both parts of the examined pile and compare displacements with the results from the dial gauges. On the basis of the excess of the collected observations, it was possible to assess the accuracy, which confirmed the usefulness of measuring techniques under study.

## 1. Introduction

Quality and capacity control procedures in civil engineering are followed during the production of building materials or structural elements and they usually consist of regular and random checks of internal strength. Tests are standardized to provide the required level of safety. In the case of industrial production, due to the uniformity of the produced materials or elements, the range of the testing can be established on the basis of the observed variability of the measured properties (strength, stiffness). Geotechnical engineering demands far more complicated control procedures [1,2] since it includes highly variable geological data, expressed by very high standard deviations of ground parameters and cyclic load [3,4]. Both the quality, expressed by continuity, integrity, and the stiffness of foundations, and the capacity measured by means of the ability to carry imposed loads, must be thoroughly checked at every stage of the construction works to form the basis for the validation of design methods and finally for the prediction of further behavior of the foundations and the subsoil caused by their displacements (settlements and horizontal movements). The inspection of foundations of a building structure is always difficult due to the large size of elements, as well as the reduced accessibility and large forces which must be applied to simulate the real conditions (foundations must carry the whole load of the building and provide a safe transmission of the loads to the ground (subsoil)). The authors’ previous experience was mostly focused on the verification of the suitability of various measuring techniques used in Static Load Testing (SLT) of foundation piles. Typical SLT is based on the constant rate of load increment applied in stages by means of hydraulic jacks. The analyzed testing procedures comprised mainly lateral and axial testing in various design situations (piles in the slope, horizontal testing of pile groups, the check of temporary piling support in the water). The results of the analyses were juxtaposed in works [5,6,7]. A more detailed analysis of geodetic method’s (terrestrial laser scanning) applicability to control displacements of hydraulic structures was given in authors’ works [8,9]. The advances in modern civil engineering make it possible to improve pile foundation techniques. This includes both the constant development of new piling technologies and also more accurate ways of analyzing pile working conditions which constitute the basic method for design and verification of adopted design solutions (as the reference test). The new developments in the field of reliability-based design also demand high accuracy of measured results [1,10,11]. Nevertheless, SLT is still considered to be the most trustworthy method of pile capacity evaluation. This method makes it possible to measure the pile head displacement under a physically applied load (axial or lateral), provided to the pile by a hydraulic jack. Despite its verified reliability, the SLT has some disadvantages. The most significant of them are the relatively long time of investigation and the potential complications while creating a suitable reaction system when the accessibility is limited due to external conditions (limited space, limited access).

### 1.1. Principles of the Tubular Pile Testing Method

**Figure 1:**
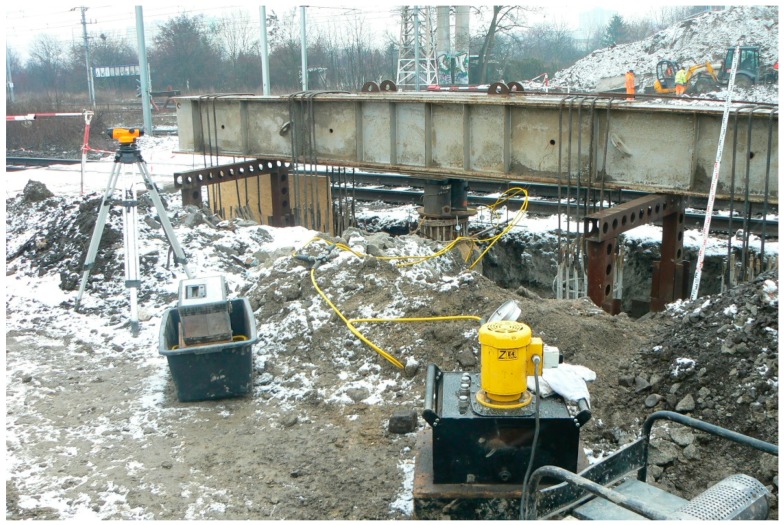
The basic idea of the SLT appliance with anchoring piles.

**Figure 2:**
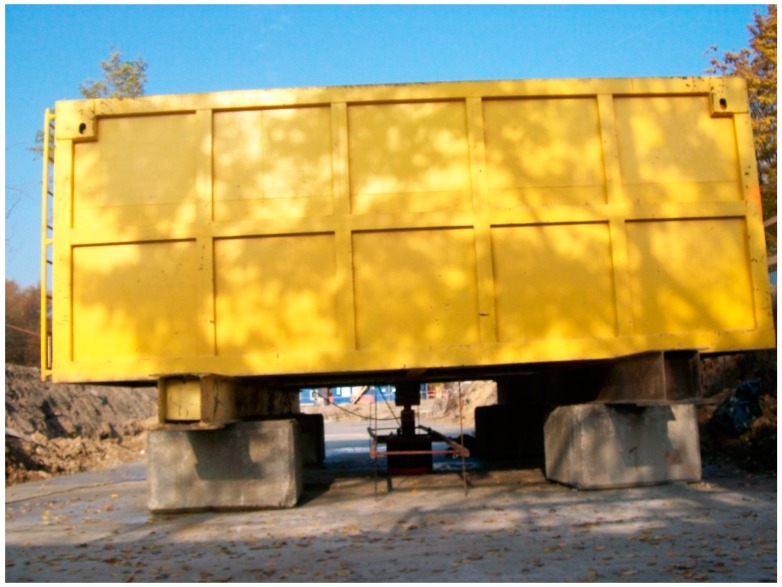
The SLT appliance with kentledge formed by water tanks.

The most frequently used structure for SLT is a so-called inverted beam, which is very expensive due to the necessity of anchoring piles construction (Figure 1) or a relevant kentledge load (Figure 2). The test is labor-intensive, technically difficult and, last but not least, seriously diminishes the randomness of the performed testing procedures (because the contractor knows which pile will be the subject of testing) [12,13]. The value of the obtained results may also be seriously affected by large deformations appearing at the last stages of loading which are crucial for pile capacity assessment. 

There are two basic issues related to displacements control in the course of SLT standard procedure. The first one is related to so-called required stabilization of settlements at every step of loading, which means that pile displacement does not increase when a constant load is applied. The stabilization is mandatory before proceeding with the next load step. The stabilization criteria differ depending on local codes of practice. Exemplary, in Poland, it is assumed that stabilization is reached when the pile head displacement does not exceed 0.05 mm within two subsequent 10 min time periods. Checking of this criteria demands very precise readings of pile displacement. In practice, various factors influencing field conditions like insolation, wind, and vibrations acting on the reference beam for dial gauges may cause higher displacement than the established limit. Instability of the reference beam could be caused by ground movements. Settlements in the vicinity of the loaded pile and possible uplift in the close distance to the anchoring piles. Other problems may be caused by the instability of the loading force in time due to deflections of the loading structure and the loss of the pressure in the hydraulic system. Due to those factors, the decision on proceeding with the next load step must be postponed and consequently, the duration of SLT mounts to unreasonable limits (dozens of hours). The application of surveying methods makes it possible to reduce the risk of the unstable reference system. 

The second main issue related to displacement control is the moment when the pile starts to “sink”—the pile’s settlement increases without the need for a rise in the applied force. The corresponding force is then called the “ultimate capacity” of the tested pile. It is assumed that the ultimate capacity is reached when the pile’s displacement is higher than 10% of its diameter. As most of the standard foundation pile technologies offer the range of diameters of 40–180 cm, one can notice that displacements corresponding to ultimate capacities must reach 40–180 mm in tests. Even if that force cannot be reached because of insufficient capacity of loading structure, it can still be evaluated from the acquired data analysis on the basis of extrapolation methods. As the precision of extrapolation is highly dependent on the performed range of the test, the force must be applied to the limits of the loading structure capacity. That moment brings many risks to the testing stuff on building site and all the operations in the vicinity of the tested pile should be limited. The surveying methods provide two benefits: the possibility of the distant control of tested pile displacements (even if the accuracy is limited, this stage of SLT has high values of displacements) and simultaneously the control of loading structure’s displacements and anchoring piles’ uplift.

The displacements of a loaded pile are usually recorded using accurate dial gauges that offer the nominal reading precision of 0.01 mm. This precision given by the manufacturer has been estimated under laboratory conditions. The real accuracy of dial gauges also depends on environmental conditions on the building site (changes in temperature, pressure, and humidity of the atmosphere) as well as the stability of the fastening of the dial gauges. The actual accuracy can be estimated up to 0.05 mm. However, it must be kept in mind that other factors determining the measured displacement such as the thermal impact on the reference beam or changes of the loading force caused by instability of pressure in the hydraulic system, may affect the measured values to a much larger extent. Large loads applied through the pile to the surrounding ground in the last stages of testing (piles are usually overloaded as compared to their working conditions) may cause excessive displacements of the soil body. Only relative displacement may be measured with the required accuracy since gauges are usually attached to a reference beam located at a close distance to the pile under test. 

**Figure 3:**
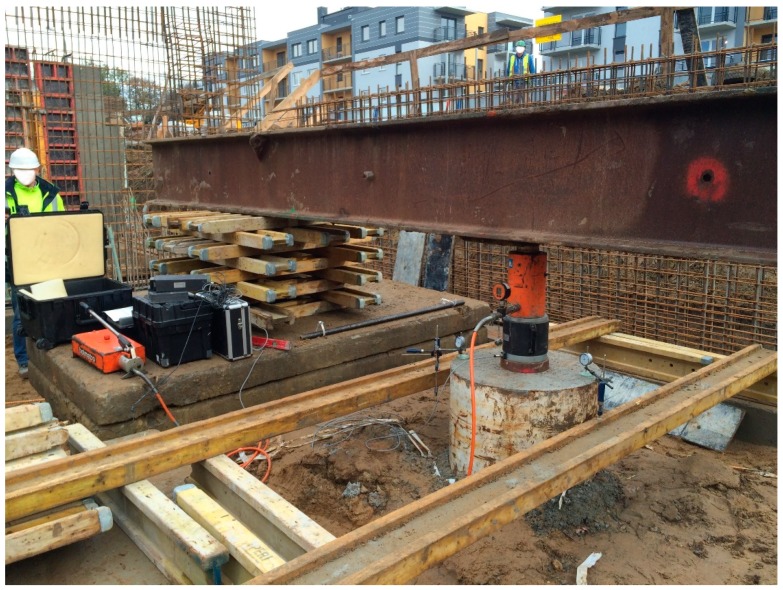
The SLT appliance with wooden reference beams.

**Figure 4:**
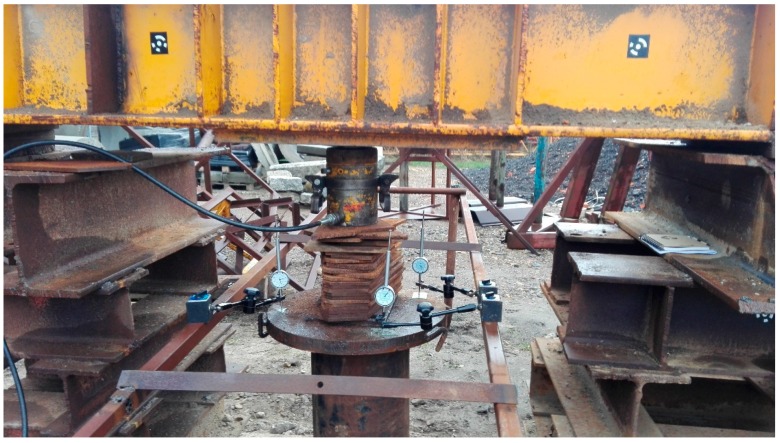
The SLT appliance with steel reference beams.

In the standard SLT procedure, the lack of reliable support for the reference beam may cause systematic errors which are very difficult to detect and which arise from an unexpected displacement of the adopted reference beam supports (Figure 3) or its own displacement caused by thermal effects (insolation) on the steel structure (Figure 4) or even the influence of the wind or water stream (when the testing appliance is built in the water, where the standard reference system for dial gauges is very difficult to establish). The application of selected geodetic methods allows the maintenance of an independent, external reference system and current verification of readings from dial gauges. 

Since 2015 Baca [14,15] have developed an original method for assessing the capacity of closed-end pipe piles. The methods presented in his works were applied to control an innovative self-balanced stand for SLT made from a closed-end double steel pipe. Field tests were conducted in order to develop the methods of evaluating the bearing capacity of the pile mobilized at its base and on its side. Preliminary comments and remarks were already presented in works of Baca [14,15,16] related to his Ph.D. research program, regarding the range of the observed displacement and the testing appliance behavior in the course of bi-directional static load testing regardless of the testing accuracy. Methods proposed by England [17] and first attempts at numerical simulation recently given by Baca in work [16] demand reliable data for the analysis. These studies consider different ways of application of the load on the pile base with regard to a different value and sign of stress transferred to the surroundings of the pile by its side surface.

### 1.2. Principles of Independent Measuring Techniques

In the course of the current research program, various geodetic displacement control techniques were applied and checked to provide a recommendation for further development. The geodetic survey constitutes the main source of information on the behavior of the pile itself, the testing structure (whose deformation is usually neglected during the test) and the ground in the vicinity of the pile under test. The crucial factors for suitability of geodetic methods are

- providing a reference system that is not associated with a testing structure; thus allowing us to determine the values of absolute displacements;
- the possibility of acquisition of many data in a short time;
- the limitation of contact between staff and the testing structure which increases the safety of the test and eliminates the accidental movement of the gauges;
- ensuring the accuracy of the measured values, however, generally, this accuracy is lower than the one obtained from gauges.

Precise leveling is the most accurate geodetic measurement technique applied to measure vertical displacements during the load test of foundation piles. Normally, the leveler is placed at a distance of several meters from the tested pile while the invar leveling rod is placed on the loaded pile, on anchor piles, and on reference points [18]. Replacing the leveling rod between the measured points in course of load test is not recommended because it requires that the staff remains in the vicinity of the burdening structure, prolongs the duration of measurement, and decreases the accuracy of readings due to the poor repeatability of the leveling rod’s putting and plumbing on the measured points. In order to permanently assemble leveling rods during the measurement, we need to have several pieces of leveling rods (including a special type with the length of 1 m) which slightly increases the cost of preparing the measurement. In recent years, precise code levelers have been applied increasingly more often, which eliminate the potential reading errors from the leveling rods. 

Constant development of measuring technologies brings an automation of displacement control and increases the accuracy of the results provided by new generations of instruments. Motorized Total Station (MTS) measurements and Terrestrial Laser Scanning (TLS) are widely implemented in engineering surveying. The question arises: can these modern measurement techniques replace or supplement the standard measuring techniques (based on dial gauges) applied to pile displacement control in the course of SLT of foundation piles and other loading tests in civil engineering (bridges, reservoirs)? In the interpretation of SLT, the main goal is to reach the pile ultimate capacity in the test or compute it by means of extrapolation methods. In most of the cases, the control of applied force is based on the measured pressure in hydraulic systems and the characteristic of the hydraulic jack. The quality and reliability of the data concerning pile displacement values are usually considered as data of secondary importance. 

The relatively high cost of MTS and laser scanners providing sufficient accuracy caused a lack of application or even tests of implementation of these instruments for SLT worldwide. The main goal of the current study is to implement available instrumentation (especially TLS) for the displacement control of the simplest self-balanced SLT stand, where the attention can be focused only on pile displacements in the course of the test. An original approach for accuracy assessment of semi-automatic measuring techniques (MTS measurement and TLS) and for vertical displacement determination on the basis of field data and point clouds from SLT (performed in the natural scale) will be proposed. 

The presented study is just the first step of research on the novel application of TLS for long-term foundation pile SLT with a perspective on further applications to control other constructions under SLT (bridges, reservoirs). The applications of laser scanning for SLT is rather a rarity and was not widely described in the literature. Some experiences were described in References [19,20] but in those cases, scanning was applied to the description of ground surface geometry around cyclically loaded piles which affect the final evaluation of the pile’s capacity. Application of terrestrial laser scanning for estimation of s the tress distribution model for elastic beam structures (multi-dimensional double-layer lattices) can be found in Reference [21]. An original algorithm for change analysis of objects loaded in a laboratory and displaced in situ (active landslide) on the basis of a point cloud from TLS was presented in Reference [22].

As the previous author’s works were focused on the reliability of the pile displacement control under SLT, the main concerns were usually based on dial gauge reference beam instability and the necessity of simultaneous control based on an independent reference system by means of surveying methods [5,6,7]. The study on the application of semi-automatic techniques (MTS measurements, TLS) is just a logical development of previous studies which makes it also possible to control the deflections (strains) of the testing appliance itself and corresponding stress and forces. The choice of the exemplary self-balanced testing appliance was motivated by its simplicity. Only the vertical displacement of the tested pile and the inner piston had to be controlled with the desired accuracy. The further studies on more sophisticated testing appliances with deflecting reaction beams and uplifting anchoring piles will be continued. 

## 2. Materials and Methods 

### 2.1. Semi-Automatic Geodetic Measuring Techniques for Determination of Displacement 

Motorized electronic total stations are more and more often being used in geodetic works. These total stations have a series of useful functions, which increase accuracy and accelerate the performance of field measurements. The most important is the automation of measurements due to the so-called ATR function (Automatic Target Recognition). The telescope of the total station is equipped with a CCD camera (consisting of pixels, which create a light-sensitive matrix) [23]. During the measurement, the beam of rays that are emitted by the total station bounces off the prism and hits one of the matrix pixels. If the central pixel was not hit, then it means that the telescope is not aimed at the center of the prism. Then, the microprocessor calculates the desired angle and adjusts the position of the telescope with the use of servomotors. The accuracy of angle measurements in the mode of automatic targeting is usually examined by the manufacturers of the total stations in laboratory conditions. The automation of measurements allows for the 3D staking out of points at the construction site, performing measurements after dark, as well as reducing the number of employees needed to perform the measurement. However, the most important application of MTS is the geodetic monitoring of the displacements of important engineering objects. Large dams, mines, slopes of landslides, or underground communication tunnels are covered by 24-h geodetic measurements, during which the precise total stations perform (at specified time intervals) automatic measurements of checkpoints, located in key spots of the structure. Examples of using MTS for the deformation monitoring of civil structures (tunnels, bridge constructions, railroads, thermal deformation of tall buildings) in the United States can be found in Reference [24]. An interesting example of integration MTS with TLS and a digital camera on a mobile platform for the monitoring and mapping of tunnel walls’ displacement has been described in Reference [25]. 

Laser scanning is a dynamically developing measurement technology, which is extensively used in various fields of science and technology. Due to the method of measuring the distance to the examined object, the scanners can be divided into three categories: pulse scanners, phase scanners, and triangulation scanners. Triangulation scanners are the most accurate, but they have a significant limitation regarding the distance range to the measured object, which usually does not exceed 1–2 m. However, there are rare models of triangulation scanners with a range up to 50 m. In the case of more distant objects, phase and pulse scanners are usually applied. The pulse scanners are characterized by the farthest range (even up to several kilometers e.g., Riegl VZ-6000), although the accuracy of measurement decreases considerably along with the increase in distance. The scanning of larger and more complex objects (especially those that are in the process of construction) requires measurements from several positions. Usually, a precise geodetic control network is established and special targets used to combine individual positions of the scanner are measured. The process of combining point clouds from different scanner positions is called registration. There are various methods to register point clouds, the description of which may be found, among others, in References [26,27,28,29]. Modern laser scanners are able to measure even up to one million points within one second. For each point, the *X*, *Y*, *Z* coordinates are recorded, as well as the parameter specifying the intensity of reflection of the laser beam. Obtained point cloud represents the actual shape of the measured object. The location of the measurement points on the object is random. The density of the cloud point is variable because on a single scanner station the scan density is defined for the most distant fragments of the object and, in addition, the scans from consecutive stations may overlap. 

Nowadays, laser scanning is very popular in forestry including the detailed mapping and classification of tree species [30,31], forest inventory [32], and the estimation of above-ground biomass of large tropical trees [33]. Most of these applications require the integration of terrestrial and aerial laser scanning, also in combination with digital photogrammetry. The statistical assessment of the digital elevation model accuracy obtained from unmanned aerial vehicles (photogrammetry) and from airborne laser scanning was presented in Reference [34]. A similar analysis for the digital terrain model was described in Reference [35]. Typical applications of airborne laser scanning in the geological investigation (extraction of surface land collapses, landslides, and fault structures) have been compiled in Reference [36]. Using laser scanning for morphological characteristics of gullies and for monitoring gully changes was given in References [37,38]. Typical applications of laser scanning in civil engineering include, among others, detailed inventory of concrete industrial heritage [39], precise 3D building modeling [40], extraction and classification of power lines [41], and monitoring tunnel deformation [42]. The control of verticality of the slender objects with the use of laser scanning is described on the example of an industrial chimney in the work [43].

### 2.2. Evaluation of Measurement Uncertainty

Each type of measurement is burdened with uncertainty which stems from the limited accuracy of the applied measuring equipment and the inaccuracy of human senses. The impact of the environment in which a given measurement is performed is also of significance. This results in measurements which are never ideally accurate and we might never reach the true value of the measured quantity. Instead, we search for the most probable value of the measured quantity. Furthermore, we strive to define the uncertainty of the measurement which carries along additional information on the dispersion of results and/or the accuracy of the applied equipment. We distinguish two basic types of uncertainty: the direct one, which is calculated on the basis of direct measurements of the searched quantity, and the indirect one, for which the searched quantity is functionally dependent to the measured quantity (it is not directly measured). When calculating the direct uncertainty, an assumption is made that the dispersion of measurements around the true value has a normal distribution. On the basis of a large number of measurements (*n* > 30), the uncertainty is expressed by means of a formula for the standard deviation of a single measurement or the standard deviation of the arithmetic mean. The uncertainty designated in this way is specified as uncertainty of type A [44]. In the case of geodetic measurements, the amount of excess observations is normally minor. While testing the precision of the measurement of angles by means of geodetic instruments in field conditions, the procedures described within the PN-ISO 17123-3:2005 standard are applied. Due to a small number of observations, this standard uses a *t*-Student distribution which is based on the number of degrees of freedom. The noted number of degrees of freedom depends on the number of performed measurement cycles (*m*) and the number of measurement series (*n*) in each cycle [45]. Upon these assumptions, the estimator of the standard deviation $u\left(\bar{X}\right)$, which corresponds to the direct uncertainty of type A in metrology, has the form of Equation (1):

(1) $$u\left(\bar{X}\right)=\sqrt{\frac{\sum_{i=1}^{m}\sum_{j=1}^{n}{\left(X_{i}-\bar{X}\right)}^{2}}{t\left(n-1\right)}},$$

where *t* is the number of measured points, *X* is the measured value (observation), and $\bar{X}$ is the value of observation.

In practice, the determination of uncertainty of type A is often strongly hampered or even impossible. For this reason, the second type of direct uncertainty was introduced, which is marked as type B: u(N). This uncertainty is marked above all for measuring devices and defines the maximum uncertainty that is possible to be obtained. In the case of digital instruments with a known limiting error (Δg) of the instrument, the uncertainty is calculated according to Equation (2):

(2) $$u\left(N\right)=\frac{\Delta g}{\sqrt{3}}$$

Limiting error represents the maximum value of an error of a measuring device during its use in line with the producer’s instructions. It is assumed that the likelihood of occurrence of a situation in which the value of the maximum error is greater than the value of an error of the arithmetical mean of measurements is insignificant. By using the previous statement, the value of the limiting error is equal to triple the value of the nominal precision indicated by the producer of a given instrument, unless the technical data state otherwise. By performing measurements by means of the same instrument, the uncertainty values of the A and B type accumulate. Thus, the final value of direct uncertainty, specified as combined uncertainty m (alternatively, standard uncertainty) is calculated according to the following Equation (3):

(3) $$m=\sqrt{u{\left(\bar{X}\right)}^{2}+u{\left(N\right)}^{2}}$$

The second type of uncertainty is indirect uncertainty, which is calculated out of the function of various variables [46]. Through the application of the error theory complex uncertainty, $u_{C}\left(H\right)$ is calculated for quantity H, on the basis of the results of direct measurements of quantities a,b,…,z (which are dependent on H but they are mutually independent) and their standard uncertainties $m_{a},m_{b},\ldots ,m_{z}$:

(4) $$u_{c}\left(H\right)=\sqrt{{\left(\frac{\partial H}{\partial a}\right)}^{2}\cdot m_{a}{}^{2}+{\left(\frac{\partial H}{\partial b}\right)}^{2}\cdot m_{b}{}^{2}+\ldots +{\left(\frac{\partial H}{\partial z}\right)}^{2}\cdot m_{z}{}^{2}}$$

where $\frac{\partial H}{\partial a}$ is the partial derivative of a function H with respect to the variable a, $\frac{\partial H}{\partial b}$ is the partial derivative of a function H with respect to the variable b, $\frac{\partial H}{\partial z}$ is the partial derivative of a function H with respect to the variable z.

### 2.3. Description of Self-Balanced Pile Capacity Testing Appliance

**Figure 5:**
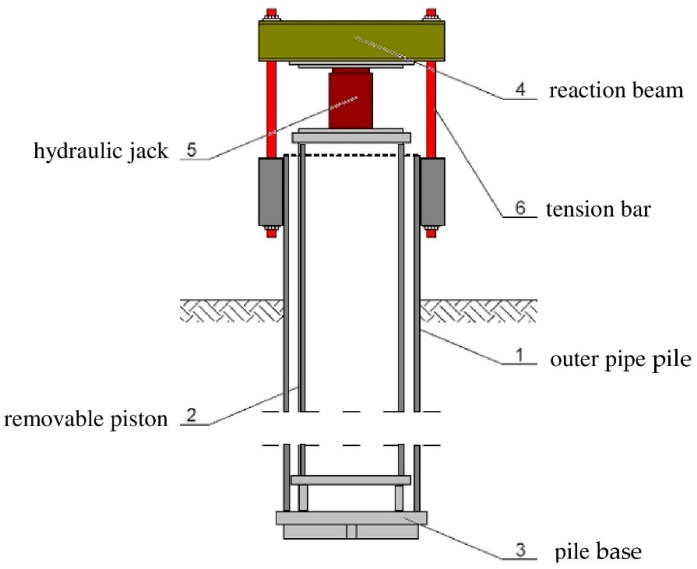
The idea of the innovative self-balanced pile testing method.

SLT, performed on a natural scale, is considered the most reliable method of pile testing, despite some of its previously mentioned disadvantages. Different approach to an SLT is presented by an Osterberg test. This investigation requires a specially designed loading device called the Osterberg cell (in the following O-cell), which is placed inside the tested pile. Pressure applied to the device creates an equal upward and downward force inside the pile. Therefore, the displacement of both sections under the applied axial load can be measured. The load applied through the O-cell allows for the simultaneous measurement of the displacement of the two sections of a pile: above and below the O-cell. Therefore, the Osterberg test allows for a separate investigation of end bearing and side shear of a pile. Despite some doubts about the comparability of results between the Osterberg test and traditional SLT, there are many works confirming the sufficient comparability between these two investigation methods [47,48]. The Osterberg test is not only a different type of an SLT, but it is also a benchmark for many testing methods which allow pile bearing capacity estimation without the necessity of creating a complex reaction system. Based on the Osterberg test assumptions, an innovative pile testing method was proposed for closed-end steel pipe piles (Figure 5) [14,15]. The force loading the pile base /3/ is transmitted from the hydraulic jack /5/ at the instrumented pile head by means of a removable piston /2/ and tension bars /6/. The hydraulic jack can be assembled on top of the piston and disassembled when the test is completed. The testing system consists of a hydraulic jack with an electric pump, reaction beam /4/, instrumentation for pile /1/ displacement control, and the reference system for geodetic measurements [6] 

### 2.4. Acquisition and Pre-Processing of Data

**Figure 6:**
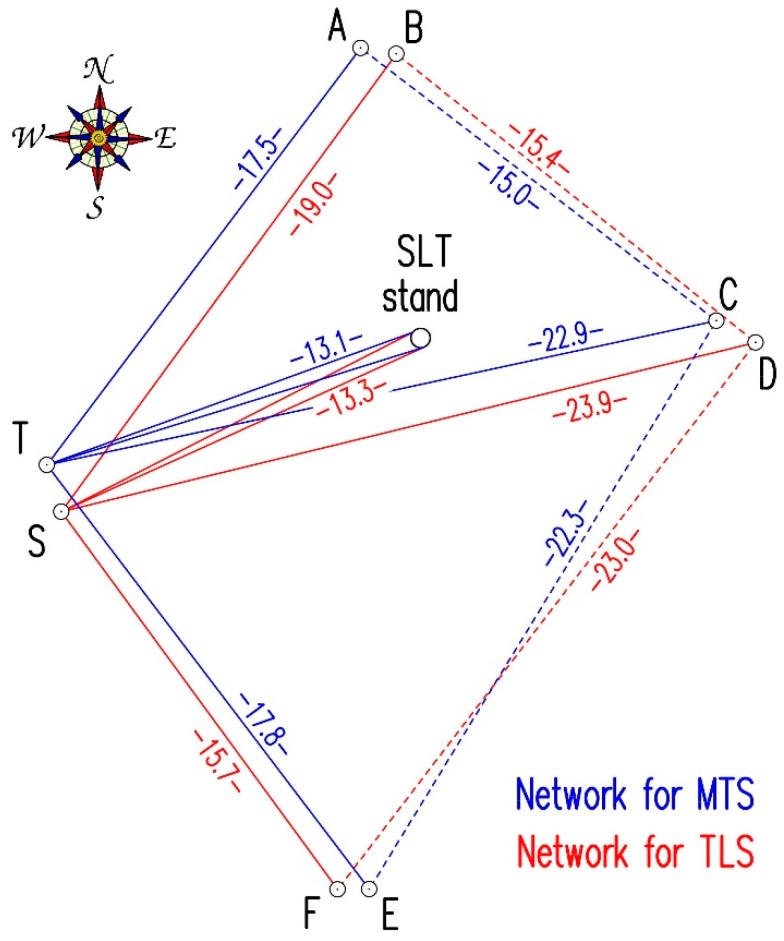
The layout of the instrument positions and measured points.

**Figure 7:**
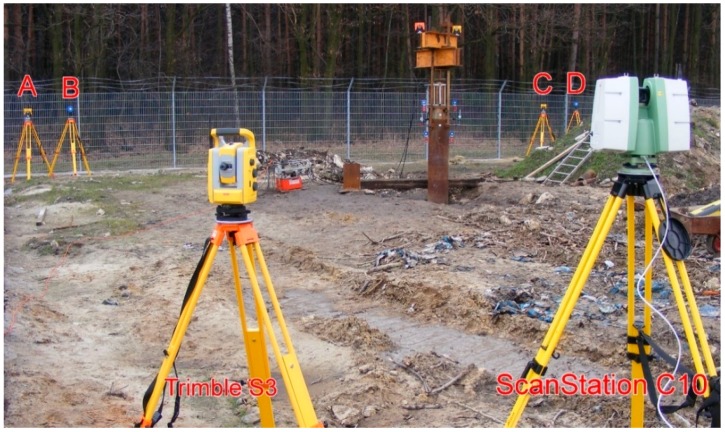
The geodetic instruments (total station Trimble S3 and laser scanner Leica ScanStation C10) with some reference points and self-balanced testing appliance.

Research works were carried out on 28 February 2015, that is, 4 weeks after pile driving. Field measurements commenced with establishing the reference frame. In case of MTS measurements, 4 reference points of the geodetic network were formed: the position of the total station T and the three points A, C, E, which were signaled by geodetic prisms. Reference frame for measurements by means of the scanner was formed by 4 separate reference points of the geodetic network: the scanner position S and three points B, D, F, which were signaled by 6-inch targets (Figure 6 and Figure 7). 

**Figure 8:**
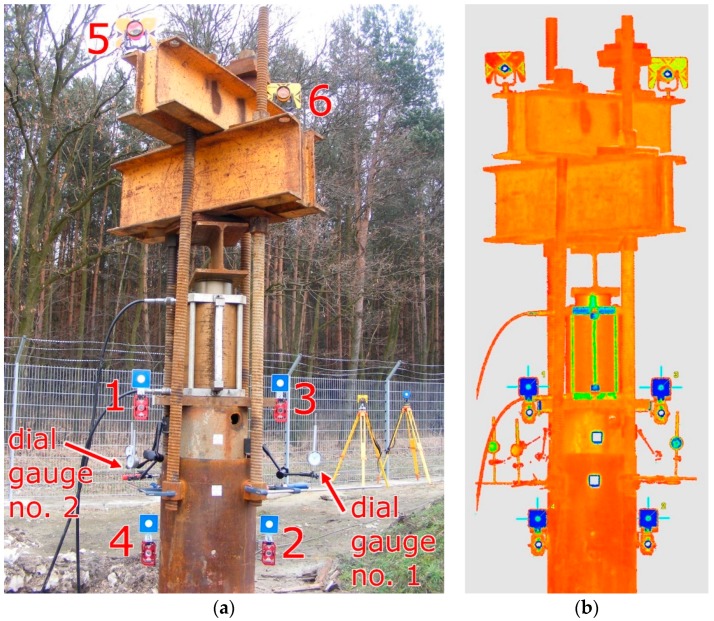
The self-balanced testing appliance: (**a**) checkpoints and dial gauges with their numbering; (**b**) point cloud colored by the intensity of reflection.

Throughout the duration of the measurements, 8 tripods were standing permanently on the reference points (with measuring instruments and prisms or targets). The chosen elements of the pile and testing appliance were represented by six checkpoints. Points no. 1 and no. 3 were mounted to the internal steel pipe and signaled with 3-inch targets and precise small prisms. Points no. 2 and no. 4 were signaled in an identical manner, mounted to the external steel pipe. Points no. 5 and no. 6 were signaled solely by geodetic prisms (Figure 8a). These two points (representing burdening structures) will not be used in the further part of the current study. Total station measurements (the polar method with trigonometric leveling) were carried out by means of the Trimble S3 total station, declared by the manufacturer to have an angle measurement accuracy of 2” and with a distance measurement accuracy (in the prism mode) equal to (2 mm + 2 ppm). All required corrections have been introduced: prism constant, temperature, pressure, scale factor equal to 1. The measurements were carried out in the automatic mode, targeting the prism (ATR). Each point was measured in two faces of the telescope (to eliminate residual instrumental errors), with triple measurement of distance in each face of the telescope. The set of measured points included reference points A, C, E, and all the checkpoints in the following order: 1, 2, 3, 4, 5, 6. The entire set of points was measured at each load step. The accepted order of the checkpoints minimized the error which could stem from the time lag between the measurements of the checkpoints representing the internal and the external pipe. Duration of a single measurement round amounted to approx. 3 min. 

Laser scanning was performed with an impulse scanner Leica ScanStation C10. The measurement accuracy of the horizontal and vertical angles given by the manufacturer amounts to 60 microradians (12.4”), while the accuracy of the reflectorless rangefinder amounts to 4 mm in a range up to 50 m. In order to shorten the time of scanning under the single measurement round, the executable script was programmed and the work of the scanner was controlled by means of a laptop. Areas, resolution, and the order of scanning were defined in the script. The measurement began with the scanning of targets B, D, F and, subsequently, the scanning of the entire testing appliance—everything with a resolution no worse than 1 mm (Figure 8b). 

The duration of a single measurement round amounted to approx. 7.5 min. Prior to starting the load test, the initial measurement was performed. The course of subsequent loading steps has been presented in Table 1. Relative displacements between the internal and the external pipe were measured traditionally by means of three dial gauges. Geodetic measurements began simultaneously with the first readings of dial gauges at each level of loading. The obtained measurement data were pre-processed. For each degree of loading and each measured point, vertical angles and spatial distances were calculated on the basis of data from the total station. In case of the scanner, for each degree of loading and each scanned target, the center of the target (vertex) was found by fitting the vertex in a point cloud of around 3.7 million points using the least square method. With the knowledge of the vertexes’ coordinates and the scanner coordinate, vertical angles and spatial distances were calculated analogically to the obtained data from the total station. 

## 3. Results

The control of the stability of reference points is necessary for the correct determination of displacement values. The first stage of the inspection included checking the stability of tripods during the whole SLT. For this purpose, the so-called search transformations method was applied separately for each control network (for the total station and for the scanner). The *x*,*y* coordinates of reference points, calculated for each consecutive loading step, were transformed isometrically (Helmert transformation with a scale factor equal to 1) into the initial *x*,*y* coordinates and the transformation errors were calculated on the basis of residuals of the reference point coordinates. These residuals indicate the compatibility between the both analyzed coordinate systems and are used to assess the accuracy of transformation by means of the mean error of coordinates *mx*, *my* and m the mean error of the position of reference points *mt*. The results of the transformations are juxtaposed in Table 2. Analysis of the values of the *mt* errors allows checking whether any of the tripods has not been hit (nudged) during the test, which occasionally happens on the construction site. The average error of transformation *mt* was equal to 0.4 mm for the total station and 0.8 mm for the scanner. The maximum error of transformation *mt* did not exceed 0.7 mm for the total station and 1.2 mm for the scanner. The obtained results indicated the stability of reference points (tripods) in the horizontal plane, which was necessary but was an insufficient condition in case of vertical displacement determination. The second stage of the inspection included checking the height changes of the reference points. During the first (initial) measurement (performed before applying the load), the known height of instrument was assumed and the heights of all reference points in the local coordinate system were calculated by the trigonometric leveling method (for MTS and separately for TLS). Next (for each loading steps) the height of the instrument was treated as unknown and the set of three values of instrument height (separately for total station and scanner) was calculated using the trigonometric leveling resection, on the basis of the known heights (from the initial measurement) of the three remaining reference points. The significant value of standard deviation *sh*, describing the dispersion of the calculated three heights of the instrument, may indicate the instability of the reference points which were targeted. The maximum value of the standard deviation *sh* of instrument’s height did not exceed 0.1 mm for the total station and 0.2 mm for the scanner (Table 2). Analysis of *sh* values does not allow us to check the stability of the instrument’s height. To control the stability of the instrument, the differences (deviations) *dh* between the assumed initial height of instrument and the average height of instrument (calculated on the basis of trigonometric leveling resection for each loading step) were calculated. The maximum absolute values of these differences *dh* did not exceed 0.1 mm for the total station and 0.4 mm for the scanner (Table 2). The value 0.0 given in the table means the absolute value is less than 0.1 mm. Based on all these results, it can be concluded that the stability of the reference frame was preserved throughout the measurement period. With this assumption, multiple measurements on the reference points (19 repetitions for each instrument) can be used to assess the accuracy of the measurement. Using Equations (1) and (2), the uncertainty of the instrument, as well as the precision of the measurement, were determined for the zenith angles and the spatial distances. Next, the standard uncertainty (Table 3) was calculated according to Equation (3).

The analysis of both types of direct uncertainty (type A and B) shows significant differences between them. Direct uncertainty of type B, which is calculated on the basis of the manufacturer’s specifications, has much bigger values than type A. This situation may result from some precaution on the manufacturer’s part, which gives the “extreme” uncertainty value while most of the produced pieces of instrument are more accurate (the “worst piece” should be within the given range). Additionally, the error of the rangefinder consists of two components: first with a constant value and second with a value that increases with the measured distance. In the case of the tested scanner (ScanStation C10), the manufacturer gives the total error for the measured distance up to 50 m. The total station is typically designed for measuring distances between several dozen and several hundred meters. The range of the distance in our case does not exceed 13.3 m to the SLT stand and 23.9 m to the furthest reference point D (Figure 6), so we can expect that the accuracy will be a little higher. The last aspect concerns the excessiveness of the obtained observations. The direct uncertainty of type B (given by manufacturer) relates to the measurement of the single point. In our case, each vertex was fitted in a cloud of over 3 million points so that the accuracy of the vertex’s coordinates should be higher. The total station measurements were also performed in two faces with triple distance measurements for each face. In the authors’ opinion, the above arguments justify the adoption of one decimal place for the results given in millimeters in further calculations in order to verify empirically the above assumptions by comparison with the results from the other tested method. 

Determination of the standard uncertainty for the zenith angles and spatial distances makes it possible to calculate the uncertainties of heights for all checkpoints. These maximum uncertainties do not exceed 0.2 mm for the total station and 1.4 mm for the scanner (Table 4). Afterward, the maximum uncertainties of the vertical displacements of checkpoints were calculated (Table 4) and did not exceed 0.3 mm for the total station and 2.0 mm for the scanner. 

**Figure 9:**
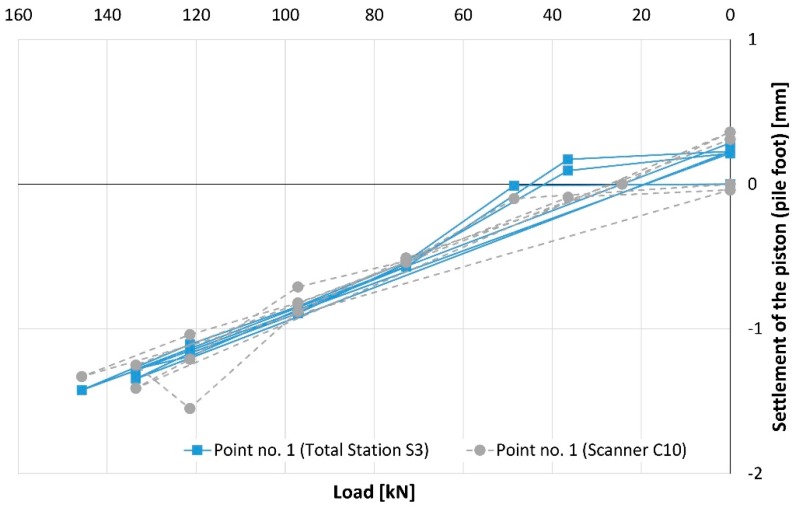
The settlement of the piston (pile foot) represented by point no. 1.

**Figure 10:**
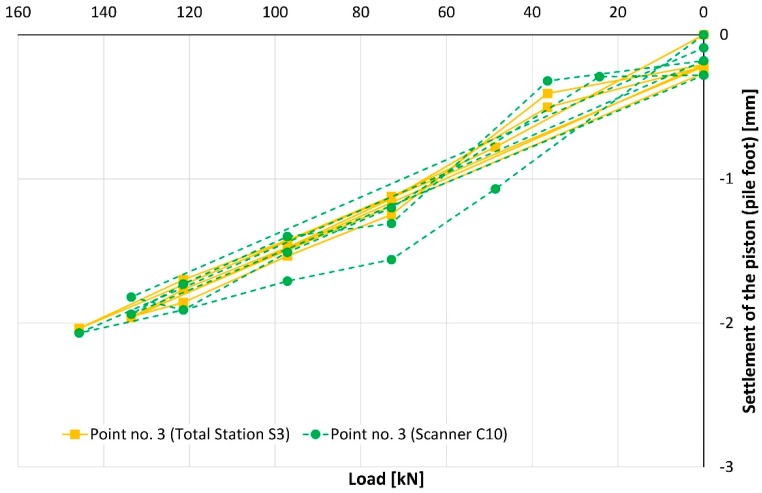
The settlement of the piston (pile foot) represented by point no. 3.

During the whole conducted SLT, a value of piston’s (pile foot) subsidence does not exceed −1.4 mm for point no. 1 (Figure 9) and −2.0 mm for point no. 3 (Figure 10), considering the total station measurements. Detailed analysis of these plots is difficult due to the overlap of lines and symbols, therefore, Table 5 was added with complementary information. 

Differences between results from MTS and TLS, as well as the simple statistics (minimum, maximum, mean and standard deviation), were calculated. In general, the values of differences correspond with the values of the indirect uncertainty of vertical displacements determination using MTS measurements. The limited amount of data do not allow for a more sophisticated statistical analysis. However, some basic remarks can be drowned out of those results. We did not observe any systematic trend concerning accuracy. Most of these discrepancies have a random character. Somewhat surprising is the high consistency between the results from the TLS and MTS (the differences do not exceed 0.4 mm). This confirms the earlier assumptions of authors stating that there exists a slightly higher scanner accuracy in this particular case than declared by the manufacturer.

**Figure 11:**
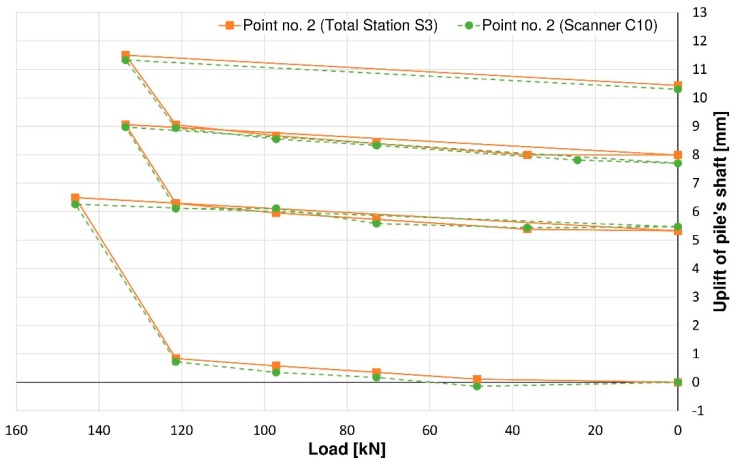
The uplift of the pile’s shaft represented by point no. 2.

**Figure 12:**
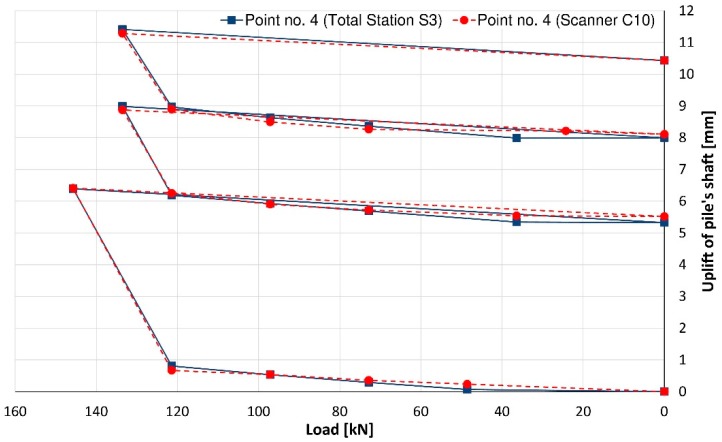
The uplift of the pile’s shaft represented by point no. 4.

**Figure 13:**
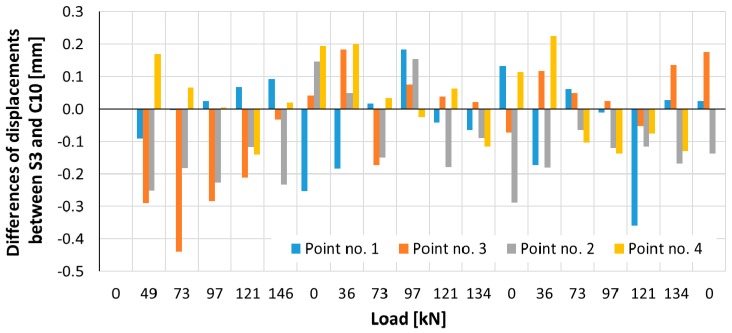
The differences of vertical displacement values between the results obtained from the total station S3 and the laser scanner C10.

**Figure 14:**
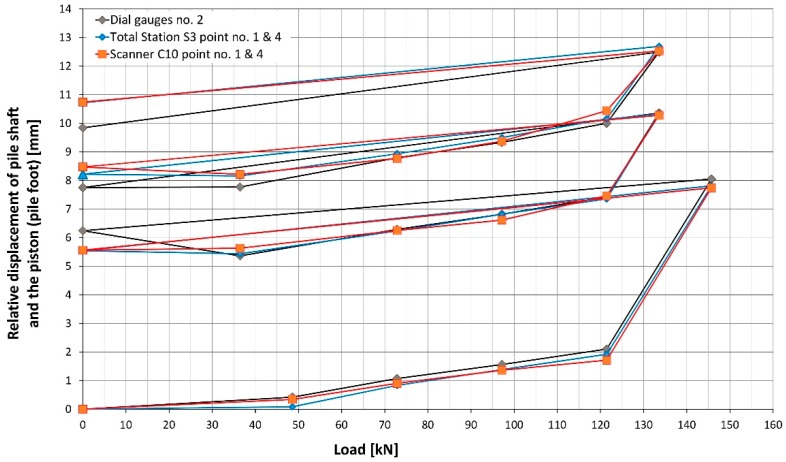
The relative displacement of the pile shaft and the piston (pile foot) calculated for a pair of checkpoints no. 1 and no. 4.

**Figure 15:**
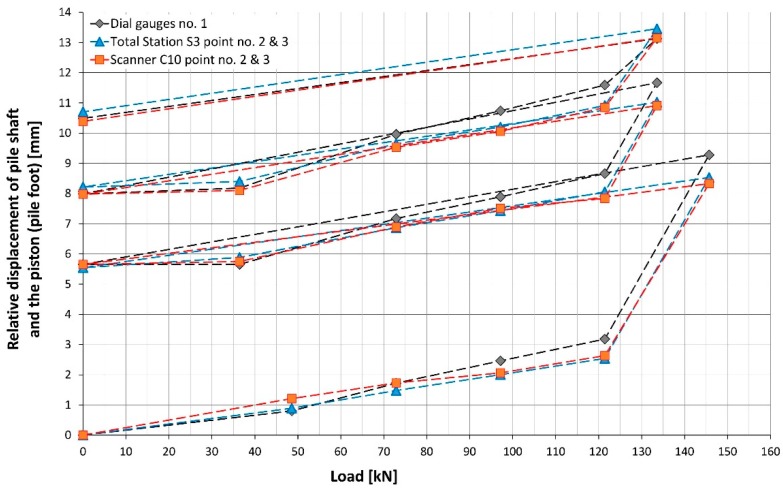
The relative displacement of pile shaft and the piston (pile foot) calculated for a pair of checkpoints no. 2 and no. 3.

Considering another pair of controlled points, the maximum vertical displacement of the pile shaft (uplift) did not exceed +11.5 mm for point no. 2 (Figure 11) and +11.4 m for point no. 4 (Figure 12) on the basis of the total station measurement. Differences of vertical displacement values between the results obtained from total station S3 and laser scanner C10 have a random character and they are presented in Figure 13. For points no. 2 and no. 4, the differences did not exceed 0.3 mm. Unfortunately, during the test, there was no external reference beam for dial gauges, so it is impossible to compare the values of absolute displacement. However, it is possible to compare the relative displacements of the piston (pile foot) with respect to the pile shaft for all measured techniques (dial gauges, MTS and TLS). These results are presented in Figure 14 and Figure 15 separately for the two pairs of checkpoints: no. 1 and no. 4 and no. 2 and no. 3. Comparing the values of relative displacements between two geodetic techniques, the differences did not exceed 0.3 mm.

However, comparing the values of relative displacements between dial gauges and two geodetic techniques, the differences are higher and reach up to 1 mm. By analyzing Figure 14 and Figure 15, we can see two characteristic situations. In the first case, the differences are significant at the first step of each loading cycle (load equal to 0 kN; compare Figure 14), afterward, they decrease and maintain the level of 0.4 mm. This situation is probably caused by the way in which the dial gauges are mounted. Usually, the mechanical connections in the loading construction loosen during unloading, however, the extreme values of displacements at the last steps of the SLT do not change significantly. The subsequent graph (Figure 15) presents the opposite (worse) situation, when the differences in displacements values increase systematically with the applied load for the first two loading cycles, which can be caused by some problems with mounting or the improper working of the dial gauge no. 1 (e.g., for reason of titling the piston). 

## 4. Discussion

Even though the idea of an Osterberg-like low-cost test of closed-end steel pipe piles (proposed by Baca) is still in a phase of a prototype, it seems that it has a market potential, at least when compared to the other time and money consuming testing procedures. It can be also used as a model test to determine soil capacity. The results of numerical simulations provided by Baca and Brząkała [16] have shown comparability between the traditional SLT and the Osterberg test. 

The above-presented geodetic techniques provide a fully independent reference system of displacement control. The analytically defined limit value of the uncertainty of vertical displacement determination in the external reference system, not related to the tested object, was 0.3 mm for the total station and 2.0 mm for the scanner. When we compare the determined displacement values of the four checkpoints calculated from both geodetic methods, the biggest differences do not exceed 0.4 mm. Therefore, it can be assumed that the value of the B type direct uncertainty for the scanner is slightly better (lower) than declared by the manufacturer, with favorable measurement conditions (short distances not exceeding 13.3 m to the SLT stand). When comparing relative displacement values obtained from dial gauges and geodetic methods, it can be noticed that the majority of differences are at the level of 0.4 mm, although sometimes even up to 1 mm. When considering these differences, additional aspects should be taken into account:

- there was some time offset which cannot be eliminated between the first and second readings from the dial gauges (averaged in further calculations) and the duration of surveying measurements (3 min for the total station and 7.5 min for the scanner);
- the location of the dial gauges and geodetic prisms/targets was slightly offset due to the limited amount of available space;
- during the unloading of the test stand and during the first reloading step, the internal pipe may tilt slightly, which may disturb the correct registration of relative displacement by dial gauges. The presented geodetic methods also enable the registration of the horizontal displacements of the loading appliance.

The most important factor for the final evaluation of pile capacity is the reliable value of pile displacement, non-influenced by systematic errors caused by weather conditions and/or large deformations of soil surrounding the pile under test. Typical reference beams used to fasten dial gauges are made of steel, bringing a relatively high risk of support instability due to thermal changes caused by insolation (Figure 4). Wooden beams (Figure 3) bring less thermal problems, but still need to be supported at a safe distance from the pile under test. In general, dial gauges with the manual or automatic registration of measured displacement may provide higher accuracy than any geodetic methods used for the test, however, the risk of dial gauge instability brings the demand for the independent method of displacement control (for post-processing analysis). Unfortunately, the application of TLS does not allow for the fast decision processing in the course of SLT.

As it was previously mentioned, in the case of the load testing of foundation piles at their final stage of loading, ensuring the accuracy of dial gauges is not a crucial factor because the range of measured displacement values highly exceed the uncertainty of the semi-automatic geodetic methods. It must be kept in mind that at the final stage of SLT, the high accuracy of displacement control is not mandatory because even when the error reaches several mm, it may result in a less than 1% error of the derived ultimate capacity. It is simply caused by high values of measured displacements and “relatively” small inaccuracies. Additionally, concerning the field conditions of SLT, some techniques based on dial gauges require the current presence of staff in the vicinity of the pile under test, which can be slightly dangerous at the final stages when large loads are applied. The conducted study proves that the use of semi-automatic methods (MTS measurement, TLS) provide a satisfactory accuracy of the results without a closer contact with excessively loaded (even overloaded) structures under test.

## 5. Conclusions

The geodetic methods offer the possibility to control a large number of points in terms of pile testing, which gives additional and reliable information over testing appliance deformation—supplementary to the standard control of the pile head (or separately base and shaft) displacement. Total station measurements (with the exception of scanning total stations) gives only a point description of the analyzed object geometry. In the case of a laser scanner, it is possible to measure specific points signaled by targets placed on the object, as well as to measure the geometry of the entire test stand described by the point cloud. Laser scanning is generally a much faster measuring technique than MTS measurements. However, in this particular application, the MTS measurements involved only 9 points and took a shorter time than laser scanning. The duration of measurements also depends on the specific instrument model (newer models are faster and can be more accurate).

Despite the limited accuracy compared to the dial gauges, the application of TLS allows for the quick control of many points, including the usually neglected deformations of the testing structure and reference beam for the dial gauges. That seems to be important when we want the results to be free from systematic errors related to climate conditions, ground movements, and the dynamic influence of works in the vicinity of the performed test. The role of surveying techniques becomes crucial when the external conditions change rapidly during the test and the movements of the ground around the pile under the test at the terminal phase of test affect the results of the traditional dial gauge measurements. 

As the application of semi-automatic geodetic measuring techniques (with a special regard to TLS) seem to be sufficiently accurate for the displacement control in course of self-balanced SLT (Figure 7) and very promising concerning the additional information about the more complex loading of construction deflections, the authors continue the research on testing appliances with reaction beams and anchoring piles in various configurations (Figure 1, Figure 2, Figure 3 and Figure 4). In these cases, the control of the reaction beam deflections may be crucial for the proper evaluation of forces acting on the anchoring piles. 

The presented research emphasizes the role of the interdisciplinary approach to geotechnical engineering. Despite the time costly post-processing of results, the TLS let us better understand the nature of the displacement of every single element of the testing appliance. The further building of the results database could help in resolving the problem of the preliminary design of the testing procedure and of the proper choice of the element strength and stiffness.

## Figures and Tables

**Table 1:** The time schedule of the performed SLT.

Step No.	Load (kN)	Time of Load Application and Delay in Starting Geodetic Meas. and First Readings of Dial Gauges	Duration of Geodetic Measurements S3/C10	Delay in the Second Reading of the Dial Gauges	Remarks
1	0	13:22 + 1 min	3 min/7.5 min	-	
2	49	13:25 + 1 min	3 min/7.5 min	+7 min	
3	73	13:34 + 1 min	3 min/7.5 min	+8 min	
4	97	13:43 + 1 min	3 min/7.5 min	+8 min	
5	121	13:51 + 1 min	3 min/7.5 min	+9 min	
6	146	14:19 + 1 min	3 min/7.5 min	+8 min	
7	0	14:30 + 1 min	3 min/7.5 min	+8 min	unloading
8	36	14:41 + 1 min	3 min/7.5 min	+25 min	battery change
9	73	15:08 + 1 min	3 min/7.5 min	+8 min	
10	97	15:17 + 1 min	3 min/7.5 min	+7 min	
11	121	15:25 + 1 min	3 min/7.5 min	+8 min	
12	134	15:43 + 1 min	3 min/7.5 min	+9 min	
13	0	15:53 + 1 min	3 min/7.5 min	+13 min	unloading
14	36	16:07 + 1 min	3 min/7.5 min	+9 min	
15	73	16:17 + 1 min	3 min/7.5 min	+7 min	
16	97	16:25 + 1 min	3 min/7.5 min	+7 min	
17	121	16:33 + 1 min	3 min/7.5 min	7 min	
18	134	16:51 + 1 min	3 min/7.5 min	+8 min	
19	0	17:00 + 1 min	3 min/7.5 min	+10 min	unloading

**Table 2:** The results of the stability control of the reference points (transformation errors for horizontal position *mt* and along both axes: *mx*, *my*, as well as the standard deviations *sh* of instrument height calculated for each step and differences of the instrument height *dh* between the initial and subsequent steps) calculated on the basis of measurements from MTS and TLS.

Load	Total Station S3					Scanner C10				
	*mx*	*my*	*mt*	*sh*	*dh*	*mx*	*my*	*mt*	*sh*	*dh*
(kN)	(mm)	(mm)	(mm)	(mm)	(mm)	(mm)	(mm)	(mm)	(mm)	(mm)
0	-	-	-	-	-	-	-	-	-	-
49	0.2	0.1	0.3	0.1	−0.1	0.5	0.3	0.6	0.1	−0.2
73	0.2	0.2	0.3	0.0	0.0	0.7	0.5	0.8	0.1	−0.2
97	0.2	0.1	0.3	0.1	−0.1	0.6	0.6	0.9	0.1	−0.2
121	0.3	0.2	0.3	0.1	−0.1	0.2	0.3	0.4	0.1	−0.3
146	0.3	0.2	0.4	0.0	−0.1	0.3	0.5	0.6	0.0	−0.3
0	0.3	0.2	0.3	0.1	−0.1	0.5	0.3	0.6	0.1	−0.3
36	0.6	0.3	0.7	0.0	−0.1	0.7	0.4	0.9	0.1	−0.2
73	0.3	0.1	0.3	0.0	−0.1	0.6	0.5	0.7	0.1	−0.3
97	0.4	0.2	0.4	0.0	0.0	0.5	0.8	0.9	0.1	−0.4
121	0.4	0.2	0.4	0.0	0.0	0.4	0.5	0.6	0.1	−0.4
134	0.1	0.4	0.4	0.0	0.0	0.5	0.6	0.8	0.2	−0.3
0	0.5	0.2	0.6	0.0	0.0	0.4	0.5	0.6	0.1	−0.3
36	0.6	0.3	0.6	0.0	0.0	0.5	0.6	0.8	0.1	−0.3
73	0.3	0.3	0.4	0.0	0.0	0.7	0.4	0.8	0.1	−0.2
97	0.3	0.1	0.3	0.0	0.0	0.7	0.6	0.9	0.1	−0.2
121	0.2	0.2	0.3	0.0	0.0	0.6	0.8	1.0	0.2	−0.2
134	0.3	0.2	0.4	0.0	0.0	0.9	0.8	1.2	0.0	−0.2
0	0.5	0.3	0.5	0.0	0.0	0.8	0.8	1.1	0.2	−0.1

**Table 3:** The values of the direct uncertainty.

Instrument	Uncertainty of B type		Uncertainty of A Type		Standard Direct Uncertainty	
	Zenith Angle	Spatial Distance	Zenith Angle	Spatial Distance	Zenith Angle	Spatial Distance
	(cc)	(mm)	(cc)	(mm)	[cc]	(mm)
Trimble S3	10.4	3.5	1.2	0.3	10.5	3.5
Leica C10	66.2	6.9	0.3	0.4	66.2	6.9

**Table 4:** The maximum values of the indirect uncertainty for the determination of the heights and vertical displacements.

Point No.	Maximum Values of Indirect Uncertainty for Determination			
	Height	Vertical Displacement	Height	Vertical Displacement
	Trimble S3		Leica C10	
	(mm)	(mm)	(mm)	(mm)
1	0.2	0.3	1.4	1.9
2	0.2	0.3	1.4	2.0
3	0.2	0.3	1.4	2.0
4	0.2	0.3	1.4	2.0

**Table 5:** The settlements of the piston (pile foot) for checkpoints no. 1 and no. 3 calculated from MTS and TLS.

Load	Settlement of the Piston (Pile Foot) (mm)					
	Point No. 1			Point No.3		
(kN)	TLS	MTS	Differences	TLS	MTS	Differences
0	0.0	0.0	0.0	0.0	0.0	0.0
49	−0.1	0.0	−0.1	−1.1	−0.8	−0.3
73	−0.5	−0.5	0.0	−1.6	−1.1	−0.4
97	−0.8	−0.8	0.0	−1.7	−1.4	−0.3
121	−1.0	−1.1	0.1	−1.9	−1.7	−0.2
146	−1.3	−1.4	0.1	−2.1	−2.0	0.0
0	0.0	0.2	−0.3	−0.2	−0.2	0.0
36	−0.1	0.1	−0.2	−0.3	−0.5	0.2
73	−0.5	−0.5	0.0	−1.3	−1.1	−0.2
97	−0.7	−0.9	0.2	−1.4	−1.5	0.1
121	−1.2	−1.2	0.0	−1.7	−1.8	0.0
134	−1.4	−1.3	−0.1	−1.9	−2.0	0.0
0	0.4	0.2	0.1	−0.3	−0.2	−0.1
24	0.0	0.2	−0.2	−0.3	−0.4	0.1
73	−0.5	−0.6	0.1	−1.2	−1.2	0.0
97	−0.9	−0.9	0.0	−1.5	−1.5	0.0
121	−1.6	−1.2	−0.4	−1.9	−1.9	−0.1
134	−1.3	−1.3	0.0	−1.8	−2.0	0.1
0	0.3	0.3	0.0	−0.1	−0.3	0.2
Min.	−1.6	−1.4	−0.4	−2.1	−2.0	−0.4
Max.	0.4	0.3	0.2	0.0	0.0	0.2
Mean	−0.6	−0.6	0.0	−1.2	−1.1	0.0
Std. dev.	0.6	0.6	0.1	0.7	0.7	0.2
